# Sexuality in Adults With ADHD: Results of an Online Survey

**DOI:** 10.3389/fpsyt.2022.868278

**Published:** 2022-05-16

**Authors:** Priscilla Gregório Hertz, Daniel Turner, Steffen Barra, Laura Biedermann, Petra Retz-Junginger, Daniel Schöttle, Wolfgang Retz

**Affiliations:** ^1^Department of Psychiatry and Psychotherapy, University Medical Center of the Johannes Gutenberg-University Mainz, Mainz, Germany; ^2^Institute for Forensic Psychology and Psychiatry, University Hospital, Homburg, Germany; ^3^Department of Psychiatry and Psychotherapy, University Medical Center Hamburg-Eppendorf (UKE), Hamburg, Germany

**Keywords:** ADHD, sexuality, sexual dysfunction, hypersexuality, sexual risk-taking, emotional dysregulation, impulsivity, oppositional symptoms

## Abstract

Establishing a secure sexual identity is a major developmental goal of an individual's transition from childhood to adolescence and the years that follow. Attention deficit/hyperactivity disorder (ADHD) as a neurodevelopmental disorder defined by the core symptoms of inattention, hyperactivity, and impulsivity, but also with emotional dysregulation, oppositional behaviors, or disorganization appearing early in life, can affect several areas of an individual's personal and social development, including sexual health. Yet, the scientific knowledge about the relationship between ADHD and sexual functioning is still scarce. Using an anonymous online survey, we compared different sexual behaviors including risky sexual behaviors, hypersexual behaviors, and sexual dysfunctions between 206 individuals with (*n* = 139) and without (*n* = 76) ADHD. Individuals with ADHD reported significantly more hypersexual behaviors than non-ADHD individuals, whereas no differences were found concerning risky sexual behaviors or sexual dysfunctions. In women with ADHD, hypersexual behaviors, sexual risk-taking as well as sexual dysfunctions were closely related to symptoms of emotional dysregulation, impulsivity, and oppositional symptoms. In men with ADHD, the associations between ADHD symptomatology and the sexuality-related measures were less clear, however, signs of emotional dysregulation seemed to be relevant as well. Since individuals with ADHD seem to be at an increased risk of some peculiarities in sexual behavior, sexuality-related issues should be routinely addressed during clinical consultations to provide more holistic treatment in order to enhance individual well-being and quality of life.

## Introduction

Establishing a secure sexual identity is a major developmental goal of an individual's transition from childhood to adolescence and in the years that follow. During puberty, not only do physiological and anatomical changes occur, but adolescents also become aware of sexual arousal, desires, and functions, and they begin to develop self-oriented and interpersonal sexual behaviors (1). Several internal and external, biological as well as environmental factors, including mental disorders, can influence emerging sexuality. Developmental disorders that appear very early in life can affect an individual's socialization and are, thus, of particular importance for the development of sexuality (2, 3).

Attention deficit/hyperactivity disorder (ADHD) is a frequent developmental disorder appearing early in life. Meta-analyses have estimated the worldwide prevalence of ADHD to be 5.3% in children and adolescents and 2.5% in adults (4, 5), with more than 50% persistence rates from childhood to adulthood (6). The core symptoms of ADHD are inattention, hyperactivity, and impulsivity. Patients with ADHD typically report problems with organization of their daily tasks and regulation of emotions (7, 8). ADHD is also related to increased risk-taking behavior (9).

Although behavioral characteristics of ADHD might also imply positive personal features that can support and sustain high functioning and flourishing life, such as cognitive dynamism, energy, divergent thinking, hyper-focus, non-conformism, adventurousness, self-acceptance and sublimation (10), diagnosis of ADHD as a developmental disorder according to DSM requires clear evidence that the symptoms interfere with, or reduce the quality of functioning in several domains of live. Indeed, ADHD is associated with a variety of risks regarding daily functioning and social adaptation (11, 12). Individuals with ADHD often show poor school performance. Their professional success is often impaired and the occupational change in a given time period is higher than in individuals without ADHD (13, 14). Furthermore, there is ample evidence that ADHD also increases the risk for violations of rules and delinquent behavior (15, 16).

ADHD is further associated with significant impairments in interpersonal relations in the family and peer context (17). Less stability in romantic relationships and less satisfaction within partnerships were found among adults with ADHD (18). Likewise, other studies found an impaired quality of romantic relationships, more maladaptive coping strategies, and less relational satisfaction within their relationships in young people with high levels of ADHD (19, 20). In addition, an increased rate of separations and divorces (18, 21, 22) and an increased risk of intimate partner violence, mainly verbal violence, has been associated with ADHD. Further, individuals with ADHD are reportedly more often victims of sexual violence (23).

Studies on the impact of ADHD on sexual development are yet scarce. In a 3-year follow-up study, no statistically significant differences in Tanner stages of sexual development were found between children aged 10 and 14 years with and without ADHD (24). Moreover, no differences regarding age of puberty onset in children with ADHD compared to children without ADHD was found and the reported age was consistent with US normative data (25). Thus, ADHD appears not to be associated with a delay or other disturbances of sexual maturation.

Concerning risk-taking behavior in the sexual domain, two studies have found a younger mean age of first sexual intercourse in young adults with ADHD compared to individuals without ADHD (26, 27). Flory et al. (26) found earlier initiation of sexual relations without sexual intercourse in participants with ADHD. A younger age at first sexual intercourse was also found in a longitudinal study on children with ADHD and healthy controls followed up for at least 13 years (11). Another study found a moderating effect of conduct problems on the association of ADHD and the age of first sexual intercourse, such that youth with high levels of ADHD symptoms and conduct problems reported the youngest age of first intercourse (28). It is important to note that the samples investigated in these studies were composed almost entirely of male participants. In contrast to findings in these studies, no differences of age at first intercourse were found among girls with and without ADHD (29, 30). However, girls with childhood ADHD engaged in oral sex at a significantly younger age and reported nearly twice as many oral sex partners than their non-ADHD peers (30).

A higher number of sexual partners among individuals with ADHD was found in several studies (11, 26). Young adults with ADHD also engaged in higher rates of other risky sexual behaviors compared with peers without ADHD, such as unprotected sex and sex while intoxicated (31–34). Accordingly, in samples of young adults with ADHD, more sexually transmitted diseases (11, 35), more partner pregnancies, and teenage parenthoods have been registered (27, 36). For example, in a cohort study based on nationwide data from Danish registers, an increased rate of teenage parenthood in individuals with ADHD compared to those without ADHD was found (37). In contrast, individuals with ADHD 25 years or older were significantly less likely to become parents than their non-ADHD counterparts (37), suggesting that there is a great need for evidence-based programs addressing teenage pregnancies in ADHD (37).

Emerging research in the past decade also highlights a high rate of co-occurring ADHD in hypersexual individuals (38). Almost a quarter of 361 hypersexual adult men seeking treatment were diagnosed with ADHD, with the vast majority (96.4%) meeting the criteria for the inattentive subtype of ADHD (39). Since problematic pornography use may be considered as a specific form of hypersexual behavior, the relationships between ADHD and problematic use of online pornography was also object of investigations in online surveys (38, 40). Results indicated that ADHD symptoms had positive moderate associations with hypersexuality in both men and women, a moderate positive association with pornography use in men, and a positive but weak association with pornography use in women (38, 40). A review of the literature on ADHD and potential sexual problems indicated that subjects with ADHD report more sexual desire, greater masturbation frequency, less sexual satisfaction, and more sexual dysfunctions than the general population (41). Although some studies showed a high prevalence of ADHD in hypersexual subjects, current findings do not clearly support the idea that hypersexuality is more frequent in individuals with ADHD (41).

Another study in an ADHD outpatient sample (42) found prevalence rates of sexual dysfunctions of 39% in male and 43% in female patients and of symptoms of any other sexual disorder in 17% of the male and in 5% of the female patients. As a comparison, in a representative study with a sample of almost 5,000 individuals of the German general population, 13.3% of the sexually active men and 17.5% of the sexually active women reported sexual dysfunctions causing marked distress (43). In spite of the high prevalence of sexual disorders, only one male ADHD patient in the sample had received a diagnosis of a sexual disorder prior to study participation (42), suggesting that sexual problems are frequently neglected in ADHD patients. In a recent study (44), sexual functioning was compared between adults with ADHD and healthy controls. Adult women with ADHD showed significantly lower scores in all Female Sexual Function Index (FSFI) domains (desire, arousal, orgasm, satisfaction, pain, and lubrication). In adult males with ADHD, the mean scores of the International Index of Erectile Function (IIEF) were lower than those of healthy controls for the subscales orgasm, erectile function, intercourse satisfaction, and overall satisfaction, but not for desire (42). One pilot study suggested several explanations for sexual dysfunctions in adults with ADHD and offered tentative routes for practical treatment approaches (45). Patients with ADHD showed significantly higher scores on single items measuring sexual dysfunctions as well as on the “anxiety/depression” scale and lower scores on the “general contentment with life” scale. However, none of the differences on a single item level between ADHD patients and controls remained significant after controlling for “anxiety/depression”. Furthermore, ADHD patients treated with methylphenidate showed stronger physical sexual arousal compared to those without methylphenidate (45).

The present study aimed to further elucidate the relation between ADHD and different aspects of sexual behavior and functioning. Given the limited number of studies and partly conflicting previous results, the present study followed an exploratory approach and thus no specific hypotheses were formulated. Specifically, we investigated risky sexual and hypersexual behaviors as well as sexual dysfunctions in women and men with ADHD in comparison to non-ADHD women and men.

## Methods

### Data Collection and Procedure

Data were collected in 2021 between July and October by means of an online survey, conducted with SoSci Survey software (46). The online survey included the German versions of internationally established questionnaires, namely the Self-Report Wender-Reimherr Adult Attention Deficit Disorder Scale [SR-WRAADDS, (47, 48)], the Sex Risk Survey [SRS, (49)], the Hypersexual Behavior Inventory [HBI-19, (50, 51)], and the Sexual Behavior Questionnaire [SBQ-G, (52, 53)] (see below). Beyond that, participants were asked questions about demographic information, ADHD diagnosis, treatment, and sexual development.

The clinical group consisted of an outpatient sample, who had been diagnosed by a team of specialized professionals including psychiatrists, psychologists and nurses and underwent an extensive diagnosing process consisting of questionnaires, interviews and neuropsychological tests at the department of psychiatry and psychotherapy of the University Medical Center of the Johannes Gutenberg University Mainz in Germany. Moreover, ADHD participants were recruited via ADHD specific Internet fora. Non-ADHD participants were recruited online as well by displaying the study call on different social media platforms and by spreading the study call through mailing lists. All information was collected anonymously, and the subjects were informed that the participation was voluntary. All participants were informed that the present study aimed at assessing sexual behaviors before study participation. Before participants were able to answer the online questionnaire they had to hit a button stating that they have read the study information and that they are willing to participate in the present study.

### Sample

In total, the survey was clicked 921 times and a total number of *N* = 344 valid cases were recorded. Of the valid cases 38 already left the survey before answering the questions about a current ADHD diagnosis. Furthermore, we excluded all participants who did not complete the whole questionnaire. Thus, our final sample consisted of 206 participants of whom 139 (89 women, 44 men, 6 diverse) were diagnosed with ADHD and 67 (44 women, 21 men, 2 diverse) had not received an ADHD diagnosis. Altogether we excluded 76 ADHD patients (35.3%) and 24 non-ADHD (26.4%) participants. The exclusion rate did not differ between ADHD and non-ADHD participants (χ^2^ = 2.34; *p* = 0.13). Furthermore, those ADHD participants who were excluded because they did not complete the whole questionnaire (*M* = 189.67; SD = 36.85) did not have a significantly higher ADHD symptom load measured with the SR-WRAADDS than those ADHD individuals who completed the whole questionnaire (*M* = 187.35; SD = 47.0). Thus, it is unlikely that non-completion of the study varied systematically depending on the ADHD symptom severity.

Since the sample size of individuals who identified themselves as diverse was too small, we could not use this group in the gender-specific analyses. However, when analyzing the whole sample, individuals who described themselves as diverse were included.

The ADHD group was on average *M* = 36.8 years old (*SD* = 10.3, Range 17 – 66), while the non-ADHD group was on average *M* = 34.3 years old (*SD* = 11.2, Range 18 – 65). Distribution of biological sex (χ^2^ = 0.355; *p* = 0.84) and age (*T* = 1.54; *p* = 0.12) did not differ significantly between the ADHD and non-ADHD participants. Of the included 139 ADHD patients 32 (23.0%) received their ADHD diagnosis at the outpatient center of the Department of Psychiatry and Psychotherapy of the University Medical Center Mainz. Of the ADHD individuals from the outpatient center 58.1% were women and they were on average 37.2 years old (SD = 10.3) and had a mean SR-WRAADDS sum score of 204.7 (SD = 30.7). In contrast, 66.7% of those ADHD individuals recruited online were women, they were on average 36.5 years old (SD = 10.4) and had a mean SR-WRAADDS sum score of 204.3 (SD = 33.9). Thus, age (*T* = *0.3*4; *p* = 0.74), gender (χ^2^ = 0.918; *p* = 0.63) and ADHD symptom load (*T* = 0.05; *p* = 0.96) did not differ between ADHD patients from the outpatient center and those recruited online.

Regarding treatment in the ADHD group, *n* = 24 (17.3%) answered that they were currently receiving no kind of treatment for their ADHD. Of those who were receiving any kind of treatment, *n* = 61 (53.0%) were currently treated psychotherapeutically, *n* = 88 (76.5%) were currently treated pharmacologically, *n* = 38 (27.3%) were receiving both psychotherapeutic and pharmacological treatment, and *n* = 15 (13.0%) patients were undergoing other sorts of treatment such as occupational therapy or neurofeedback. Since no further differentiation of the substances used in the pharmacological treatment was made, we did not consider this information for further statistical analyses.

Comparisons of the mean sum scores of both ADHD and non-ADHD groups on the SR-WRAADDS showed that individuals with ADHD had significantly higher scores on all subscales (see Table 1). This accounted for women as well as for men.

**Table 1:** Comparison between mean SR-WRAADDS sum score and subscale scores in individuals with and without ADHD.

	**Whole sample**			**Women**			**Men**		
	**ADHD**	**HCs**	**Sig**.	**ADHD**	**HCs**	**Sig**.	**ADHD**	**HCs**	**Sig**.
	**(*n* = 139)**	**(*n* = 67)**		**(*n* = 89)**	**(*n* = 42)**		**(*n* = 44)**	**(*n* = 21)**	
SR-WRAADDS sum score	205.45	149.82	T = 8.05	206.35	149.57	T = 8.22	203.27	148.10	T = 5.17
	SD = 30.94	SD = 52.32	*p* < 0.001	SD = 31.39	SD = 46.60	*p* < 0.001	SD = 28.24	SD = 58.17	*p* < 0.001
Attention difficulties	24.40	17.13	T = 8.27	24.27	17.31	T = 7.86	24.82	16.33	T = 6.71
	SD = 3.52	SD = 6.77	*p* < 0.001	SD = 3.75	SD = 6.35	*p* < 0.001	SD = 3.01	SD = 7.21	*p* < 0.001
Hyperactivity	10.74	7.84	T = 6.06	10.92	7.52	T = 6.56	10.16	8.19	T = 2.32
	SD = 2.72	SD = 3.44	*p* < 0.001	SD = 2.61	SD = 3.09	*p* < 0.001	SD = 2.92	SD = 3.72	*p = 0.0*2
Temper	10.14	7.57	T = 4.95	10.35	7.31	T = 4.93	9.91	7.86	T = 2.39
	SD. = 3.12	SD = 3.66	*p* < 0.001	SD = 3.20	SD = 3.47	*p* < 0.001	SD = 2.88	SD = 3.90	*p* = 0.02
Affective lability	20.29	14.79	T = 7.31	20.55	14.74	T = 7.96	19.68	14.57	T = 4.37
	SD = 3.11	SD= 5.76	*p* < 0.001	SD = 3.14	SD = 5.18	*p* < 0.001	SD = 3.05	SD = 6.42	*p* < 0.001
Emotional over-reactivity	15.55	12.16	T = 5.42	15.88	12.71	T = 4.76	15.0	10.81	T = 4.36
	SD = 3.20	SD = 4.60	*p* < 0.001	SD = 3.04	SD = 4.45	*p* < 0.001	SD = 3.26	SD = 4.31	*p* < 0.001
Disorganization	23.25	17.13	T = 6.12	23.28	17.0	T = 6.12	23.27	16.86	T = 3.96
	SD = 4.73	SD = 7.50	*p* < 0.001	SD = 4.65	SD = 6.95	*p* < 0.001	SD = 4.80	SD = 8.24	*p* < 0.001
Impulsivity	17.87	11.63	T = 9.24	18.38	11.52	T = 8.82	16.91	11.67	T = 4.73
	SD = 3.97	SD = 4.80	*p* < 0.001	SD = 3.99	SD = 4.48	*p* < 0.001	SD = 3.84	SD = 4.83	*p* < 0.001
Oppositional symptoms	34.0	24.88	T = 6.29	33.38	23.76	T = 5.99	34.93	27.48	T = 3.18
	SD = 7.80	SD = 10.57	*p* < 0.001	SD = 7.95	SD = 9.79	*p* < 0.001	SD = 7.20	SD = 11.63	*p* = 0.002
Academic problems	12.48	8.82	T = 6.29	12.90	8.95	T = 5.58	11.61	8.38	T = 2.97
	SD = 3.83	SD = 4.08	*p* < 0.001	SD = 3.68	SD = 3.99	*p* < 0.001	SD = 4.02	SD = 4.28	*p* = 0.004
Social attitudes	36.71	27.87	T = 5.64	36.44	28.74	T = 4.28	36.98	25.95	T = 4.37
	SD = 8.86	SD = 11.27	*p* < 0.001	SD = 8.60	SD = 11.48	*p* < 0.001	SD = 9.18	SD = 10.21	*p* < 0.001

*HCs, healthy controls*.

### Measures

#### Self-Report Wender-Reimherr Adult Attention Deficit Disorder Scale (SR-WRAADDS)

The Self-Report Wender-Reimherr Adult Attention Deficit Disorder Scale [SR-WRAADDS; (47, 48)] was developed based on the interviewer-administered WRAADDS (54, 55), in order to assess ADHD symptomatology via self-report on seven domains related to the ADHD: attention difficulties, hyperactivity/ restlessness, temper, affective lability, emotional overreactivity, disorganization and impulsivity (47). The scale used in the present study also includes three other domains of symptoms associated with ADHD, namely oppositional symptoms, academic problems, and social attitudes. The psychometric properties of the SR-WRAADDS support its use in research and clinical practice (47, 48, 56, 57). The SR-WRAADDS showed high correlations with the WRAADDS in an ADHD sample and every domain demonstrated discriminatory validity comparing adults with ADHD with healthy controls (47). In a German validation study, the correlation between the WRAADDS and the SR-WRAADDS was significant and the retest reliability was high. Further results showed an adequate discriminatory validity and a satisfactory to high internal consistency for the individual subscales (48).

#### Sexual Risk Survey (SRS)

The Sexual Risk Survey [SRS; (49)] is a questionnaire developed for measuring risky sexual behavior. The SRS emphasizes some specific aspects of sexual risk taking such as sex with an uncommitted partner, impulsive sex and risky anal sex, as referring to unprotected anal sex, anal fisting and oral anal practices. Study results showed a good internal consistency and test-retest reliability. The SRS also demonstrated evidence of convergent and concurrent validity by its relationships with reported number of sexual partners and history of infidelity as well as measures of sensation seeking, sexual desire, substance use, sexual excitation and inhibition, and sexual health consequences (49). For the present study, the English version of the SRS was first translated from English to German. In a second step, another person of our research team, who was also fluent in both languages, translated the German version back to English. In a third step, the original English version and the English translated version were compared. In case of differences in meaning, adjustments were made after discussing them in the team and finding an agreement, which was unproblematic in every case.

#### Hypersexual Behavior Inventory (HBI-19)

The HBI-19 (50) is a self-report measure of hypersexual behavior. The inventory includes 3 s-order factors of (1) attempting to control sexual thoughts, feelings, and behaviors, (2) attempting to cope with unwanted emotions and life stressors, and (3) experiencing undesirable consequences related to problematic sexual behaviors. Scores range from 19 to 95 with higher scores indicating higher levels of hypersexuality and are considered as being clinically relevant at 53 or higher (50). In the German validation study (51), the psychometric properties of the German version of the HBI-19 were investigated as part of an online survey in a representative sample consisting of 1,749 men and women. The questionnaire showed good reliability and validity. A confirmatory factor analysis supported the 3-factor structure of the original English version (51).

#### Sexual Behavior Questionnaire-German Version (SBQ-G)

The German version of the Sexual Behavior Questionnaire [SBQ-G, (52, 53, 58)] is a short self-rating instrument based on the ICD-10 classification of sexual dysfunctions and covers different domains of sexual function, such as libido, sexual arousal, and sexual satisfaction. Findings on the original version (52) proved the clinical validity of this questionnaire. In previous studies (53, 58), retest reliability for the global index was good and the face validity was high. Sexual arousal and ability to enjoy sex showed the lowest retest reliability. In general, reception of the SBQ-G was favorable. Thus, the SBQ-G has been recommended as a clinical instrument with high practicability and satisfactory retest reliability (52, 53, 58).

### Statistical Analyses

We analyzed the sample by comparing demographic data and information concerning ADHD. The analyses of the present study were of exploratory nature and aimed to contribute to the generation of further hypotheses. The mean scores of both groups on the SR-WRAADDS were compared using *T*-tests. First, ADHD and HC (healthy control) groups were compared regarding their sexual orientation and sexual experience by means of *Chi*^ 2^-tests. Further, we compared differences in the mean frequency of risky sexual behavior in the two groups for the whole sample and separated by the gender binary system (women/men). Although we implemented the non-binary system in the survey, the sample sizes of the other categories were too small to be included in further statistical analyses. Correlations were further calculated between ADHD symptomatology measured with the SR-WRAADDS and sexual risk behavior measured with the SRS. Moreover, we compared differences in the mean frequency of hypersexual behaviors using the HBI-19 in the two groups within the whole sample, and separately for women and men using *T*-tests. We also calculated correlations between ADHD symptoms and hypersexual behavior using the SR-WRAADDS sum scores and the HBI-19 sum scores and its three factors (control attempts, sex as coping strategy, negative consequences). Additionally, we calculated a backward step binary logistic regression using the SR-WRAADDS subscale scores as predictors and the HBI-19 cut-off value as dependent variable, both in the whole sample and separately in women and men with ADHD, in order to examine the predictive power of the ADHD symptoms on hypersexual behaviors. Further, we analyzed the frequency of sexual dysfunctions in the ADHD and non-ADHD groups such as erectile dysfunction, delayed ejaculation, early ejaculation, and low orgasm satisfaction within men and low orgasm satisfaction, sexual pain, and failure to achieve orgasm within women using *Chi*^ 2^-tests. Finally, we calculated correlations between ADHD symptoms using the SR-WRAADDS [WR-SR, (48)] and sexual dysfunctions using the SBQ-G within men and women with ADHD. The statistical significance value was set on a threshold of *p* < 0.05 for all the statistical analyses. Because we calculated multiple group comparisons for each construct, we corrected for multiple testing where appropriate using the false discovery rate [FDR, (59)]. Although discussed controversially, it has been recently suggested that in exploratory analysis alpha-level adjustment is less necessary (60) and thus we were less strict in rejecting results that were not statistically significant any longer after correcting for multiple testing. Nevertheless, we have marked all results that were not significant or that were still significant, respectively, after correcting for multiple testing. All statistical analyses were performed using SPSS 26 (IBM).

## Results

### General Sexual Behaviors and Orientation

On average, individuals with ADHD were not significantly younger at the time of their first sexual experiences. However, when comparing women and men separately, a younger age was found in women with ADHD than in non-ADHD women, while no differences occurred when comparing male participants (see Table 2). No significant difference occurred between women and men with ADHD (*T* = −1.54, *p* = 0.13). Furthermore, no differences were found concerning the number of ADHD and non-ADHD participants who reported that their first sexual experience was an experience of sexual abuse (see Table 2). However, women with ADHD (*n* = 22, 24.7%) reported about an experience of sexual abuse significantly more often than men with ADHD (*n* = 2, 4.5%) (χ^2^ = 8.10, *p* = 0.004), whereas this significant pattern was not found in individuals without ADHD (women: *n* = 6, 14.3%, men: *n* = 0, χ^2^ = 3.3; *p* = 0.07).

**Table 2:** Sexual orientation and sexual experiences in individuals with and without ADHD.

	**Whole sample**			**Women**			**Men**		
	**ADHD**	**HCs**	**Sig**.	**ADHD**	**HCs**	**Sig**.	**ADHD**	**HCs**	**Sig**.
	**(*n* = 139)**	**(*n* = 67)**		**(*n* = 89)**	**(*n* = 42)**		**(*n* = 44)**	**(*n* = 21)**	
Age at first sexual experience	15.3 years	17.1 years	T = −1.73	14.3 years	17.0 years	T = −3.02	15.8 years	16.5 years	T = −0.37
	SD = 7.25	SD = 6.92	*p* = 0.09	SD = 3.73	SD = 3.73	*p* = 0.003	SD = 5.89	SD = 7.30	*p* = 0.71
Sexually abusive experience	25 (18%)	6 (9%)	χ^2^ = 2.88	22 (24.7%)	6 (14.3%)	χ^2^ = 1.85	2 (4.5%)	0	χ^2^ = 0.99
			*p* = 0.09			*p* = 0.17			*p* = 0.32
Sexual orientation			χ^2^ = 3.90			χ^2^ = 3.77			χ^2^ = 1.22
			*p* = 0.27			*p* = 0.29			*p* = 0.54
Heterosexual	96 (69.1%)	51 (76.1%)		60 (67.4%)	31 (73.8%)		35 (79.5%)	19 (90.5%)	
Homosexual	3 (2.2%)	3 (4.5%)		1 (1.1%)	2 (4.8%)		0	0	
Bisexual	23 (16.5%)	5 (7.5%)		18 (20.2%)	4 (9.5%)		4 (9.1%)	1 (4.8%)	
Unsure/other	17 (12.2%)	8 (11.9%)		10 (11.2%)	5 (11.9%)		5 (11.4%)	1 (4.8%)	
Heterosexual experiences			χ^2^ = 0.59			n/A			χ^2^ = 0.30
			*p* = 0.45						*p* = 0.59
Yes	137 (98.6%)	65 (97.0%)		89 (100%)	42 (100%)		43 (97.7%)	20 (95.2%)	
No	2 (1.4%)	2 (3.0%)		0	0		1 (2.3%)	1 (4.8%)	
Homosexual experiences			χ^2^ = 11.12			χ^2^ = 6.56			χ^2^ = 3.87
			*p <0.0*01			*p* =0.01			*p* = 0.05
Yes	67 (48.2%)	16 (23.9%)		51 (57.3%)	14 (33.3%)		11 (25.0%)	1 (4.8%)	
No	72 (51.8%)	51 (76.1%)		38 (42.7%)	28 (66.7%)		33 (75.0%)	20 (95.2%)	

*HCs, healthy controls*.

No significant differences were found concerning self-reported sexual orientation between individuals diagnosed with ADHD and those without ADHD (see Table 2). Participants specified being heterosexual most frequently, followed by a bisexual orientation. Only a minority reported a homosexual orientation. When comparing the number of individuals with previous heterosexual and homosexual experiences, we found that almost all participants regardless of an ADHD diagnosis described previous heterosexual experiences. However, significantly more individuals with an ADHD diagnosis reported about previous homosexual experiences than individuals without an ADHD diagnosis. This accounted for women and for men, whereby this difference was even more pronounced in women with ADHD (see Table 2).

### Hypersexual Behaviors

Individuals with ADHD had significantly higher HBI-19 total scores and significantly higher scores on the HBI-19 subscales (coping, consequences, control) compared to individuals without ADHD. However, no significant difference was found in the number of participants in the two groups who were at or above the pre-defined HBI-19 cut-off value of 53, which allows to classify an individual as hypersexual (see Table 3). When comparing women and men separately, we found that both women and men with ADHD had significantly higher HBI-19 total scores and significantly higher scores in the HBI-19 control scale than their non-ADHD counterparts, whereas no significant differences could be observed in the other two HBI-19 subscales.

**Table 3:** Comparisons of hypersexual behaviors between individuals with ADHD and without ADHD using the HBI-19.

	**Whole sample**			**Women**			**Men**		
**Hypersexual Behavior Inventory (HBI-19)**	**ADHD**	**HCs**	**Sig**.	**ADHD**	**HCs**	**Sig**.	**ADHD**	**HCs**	**Sig**.
	**(*n* = 139) *M* (SD)**	**(*n* = 67) *M* (SD)**		**(*n* = 89)**	**(*n* = 42)**		**(*n* = 44)**	**(*n* = 21)**	
HBI sum	40.43 (17.4)	32.52 (14.61)	T = 3.20	35.58 (14.41)	29.10 (12.14)	T = 2.53	50.43 (19.02)	40.86 (17.01)	T = 1.96
			***p*** **=** **0.002**			***p*** **=** **0.01**			*p* = 0.05
HBI coping	16.71 (7.44)	14.05 (6.88)	T = 2.46	15.25 (6.91)	12.98 (6.19)	T = 1.81	19.52 (7.74)	16.95 (7.83)	T = 1.25
			***p*** **=** **0.02**			*p* = 0.07			*p* = 0.22
HBI consequences	7.04 (3.75)	5.79 (2.98)	T = 2.39	5.84 (2.73)	5.0 (2.37)	T = 1.72	9.46 (4.27)	7.71 (3.45)	T = 1.63
			***p*** **=** **0.02**			*p* = 0.09			*p* = 0.11
HBI control	16.68 (8.43)	12.69 (6.08)	T = 3.88	14.49 (7.06)	11.12 (4.52)	T = 3.30	21.45 (9.11)	16.19 (7.53)	T = 2.30
			***p*** **<** **0.001**			***p*** **<** **0.001**			*p* = 0.03
Above cut off	34 (24.5%)	10 (14.9%)	χ^2^ = 2.45	13 (14.6%)	4 (9.5%)	χ^2^ = 0.65	20 (45.5%)	6 (28.6%)	χ^2^ = 1.69
			*p* = 0.12			*p* = 0.42			*p* = 0.19

*HCs, healthy controls. Bold values mean statistical significance after correcting for multiple testing*.

Finally, it could be observed that men with ADHD had significantly higher HBI-19 total scores (*T* = −4.57; *p* < 0.001) and significantly higher scores in all subscales (Coping: *p* = 0.002; Consequences: *p* < 0.001; Control: *p* < 0.001) than women with ADHD. Furthermore, significantly more men with ADHD were above the HBI-19 cut-off value than women with ADHD (χ^2^ = 15.02; *p* < 0.001) and could thus be classified as hypersexual.

To determine which of the SR-WRAADDS symptom scales were related to hypersexual behaviors, we calculated a correlation analysis between the subscales and sum scores of the SR-WRAADDS and the HBI-19 for the whole sample and separately for women and men with ADHD. Using the whole sample we found significant correlations between the SR-WRAADDS sum score (*r* = 0.25; *p* = 0.004), the subscales temper (*r* = 0.20; *p* = 0.02), disorganization (*r* = 0.19; *p* = 0.03), oppositional symptoms (*r* = 0.29; *p* = 0.001), and social attitudes (*r* = 0.27; *p* = 0.001) on the one side and the HBI-19 sum score on the other side. The HBI-19 control subscale was significantly associated with the SR-WRAADDS sum score (*r* = 0.28; *p* = 0.001), the subscales temper (*r* = 0.21; *p* = 0.01), emotional over-reactivity (*r* = 0.17; *p* = 0.05), disorganization (*r* = 0.22; *p* = 0.01), oppositional symptoms (*r* = 0.26; *p* = 0.002), and social attitudes (*r* =0.31; *p* < 0.001). The HBI-19 consequences subscale was also significantly associated with the SR-WRAADDS sum score (*r* = 0.19; *p* = 0.03), with the subscales disorganization (*r* = 0.18; *p* = 0.04), oppositional symptoms (*r* = 0.21; *p* = 0.02), and social attitudes (*r* = 0.27; *p* = 0.002). Finally, the HBI-19 coping subscale was significantly correlated with the SR-WRAADDS sum score (*r* = 0.17; *p* = 0.04) and with the subscale oppositional symptoms (*r* = 0.28; *p* = 0.001).

In women, the SR-WRAADDS sum score as well as the subscales temper, affective lability, impulsivity, and oppositional symptoms significantly correlated with the HBI-19 sum score and with all three HBI-19 subscales (see Table 4). In contrast, in men only the SR-WRAADDS subscale disorganization significantly correlated with the HBI-19 control subscale, and the SR-WRAADDS social attitudes subscale was significantly associated with the HBI sum score as well as the HBI-19 consequences and control subscales (see Table 4).

**Table 4:** Associations between ADHD symptoms and hypersexual behaviors in women and men with ADHD using the SR-WRAADDS and the HBI-19.

	**HBI sum score**	**HBI Coping**	**HBI Consequences**	**HBI Control**
**Women**				
SR-WRAADDS sum score	0.35^**^	0.27^*^	0.26^*^	0.35^**^
Attention difficulties	0.05	−0.08	0.10	0.15
Hyperactivity	0.10	0.11	0.02	0.09
Temper	0.32^**^	0.26^*^	0.22^*^	0.31^**^
Affective lability	0.33^**^	0.27^*^	0.21^*^	0.34^**^
Emotional over-reactivity	0.26^*^	0.20	0.10	0.28^**^
Disorganization	0.17	0.10	0.14	0.19
Impulsivity	0.33^**^	0.27^**^	0.23^*^	0.30^**^
Oppositional symptoms	0.37^**^	0.37^**^	0.24^*^	0.29^**^
Academic problems	0.16	0.06	0.12	0.21^*^
Social attitudes	0.24^*^	0.16	0.23^*^	0.25^*^
**Men**				
SR-WRAADDS sum score	0.21	0.08	0.21	0.27
Attention difficulties	−0.04	−0.21	0.04	0.08
Hyperactivity	−0.18	0	−0.27	−0.25
Temper	0.08	0.01	0.11	0.12
Affective lability	0.05	−0.07	0.15	0.09
Emotional over-reactivity	0.19	0.15	0.17	0.19
Disorganization	0.27	0.14	0.27	0.32^*^
Impulsivity	−0.09	−0.06	−0.11	−0.09
Oppositional symptoms	0.12	0.07	0.07	0.15
Academic problems	0.15	0.06	0.17	0.19
Social attitudes	0.35^*^	0.14	0.35^*^	0.44^**^

* *p = 0.05*;

** *p < 0.01; Bold values mean statistical significance after correcting for multiple testing*.

Furthermore, we calculated a backward step binary logistic regression with the SR-WRAADDS subscale scores as predictors and the HBI-19 cut-off as dependent variable separately for the whole sample and for women and men with ADHD. In the whole sample, hyperactivity was a negative predictor (β = 0.83; 95% CI 0.70 – 0.98; *p* = 0.03), while oppositional symptoms (β = 1.08; 95% CI 1.01 – 1.16; *p* = 0.05) and social attitudes (β = 1.06; 95% CI 1.0 – 1.12; *p* = 0.05) were positive predictors of hypersexuality. The final regression model explained 18% of the variance of the HBI-19 cut-off value and 76.3% of all participants could be correctly classified as hypersexual or not. In women, only the impulsivity subscale score was a significant predictor (β = 1.30; 95% CI 1.06 – 1.60; *p* = 0.01), while all other variables were not included in the final regression model. The final regression model explained 16.2% of the variance of the HBI-19 cut-off value and 85.4% of the female ADHD individuals were correctly classified as being below or above the HBI-19 cut-off. In men, the subscale affective lability was negatively associated with the HBI-19 cut-off (β = 0.78; 95% CI 0.61 – 1.0; *p* = 0.05), while the social attitudes subscale was positively associated with the HBI cut-off (β = 1.13; 95% CI 1.03 – 1.24; *p* = 0.01). The final regression model explained 26.2% of the variance of the HBI-19 cut-off value and 63.6% of the male ADHD individuals were correctly classified as being below or above the HBI-19 cut-off.

### Sexual Risk Taking

No group differences were found in the comparison of risky sexual behaviors using the SRS. However, men with ADHD reported more frequently about leaving a social event with a person they have just met than men without ADHD (see Table 5). When comparing men and women with ADHD on the sexual risk variables, women with ADHD reported more frequently about using alcohol or drugs before or during sex than men with ADHD (*T* = 2.08, *p* = 0.04). All other variables did not differ significantly between men and women with ADHD.

**Table 5:** Comparisons of sexual risk behaviors between individuals with and without ADHD using the SRS.

	**Whole sample**			**Women**			**Men**		
**Sex Risk Survey (SRS)**	**ADHD**	**HCs**	**T**	**ADHD**	**HCs**	**T**	**ADHD**	**HCs**	**T**
	**(*n* = 139)**	**(*n* = 67)**		**(*n* = 89)**	**(*n* = 42)**		**(*n* = 44)**	**(*n* = 21)**	
1. How many partners have you engaged	1.77	2.32	–0.82	1.73	2.83	–0.97	1.39	1.45	–0.08
in sexual behavior with but not had sex with?	(3.73)	(5.82)		(3.41)	(6.96)		(2.79)	(2.78)	
2. How many times have you left a social	0.99	0.82	0.30	1.13	1.21	–0.09	0.75	0.10	**2.09** ^*^
event with someone you just met?	(2.82)	(5.22)		(3.22)	(6.48)		(2.01)	(0.31)	
3. How many times have you “hooked up” but	1.47	1.31	0.22	1.52	1.67	–0.15	1.14	0.65	0.63
not had sex with someone you didn't know or didn't know well?	(4.46)	(5.36)		(4.91)	(6.52)		(3.15)	(2.06)	
4. How many times have you gone out with the	3.63	2.51	–0.82	2.42	1.50	0.47	6.50	5.00	0.25
intent of “hooking up” and having sex with someone?	(15.56)	(13.55)		(11.55)	(7.11)		(22.17)	(22.36)	
5. How many times have you had an	1.96	1.25	0.79	2.33	1.71	0.45	1.39	0.40	1.76
unexpected and unanticipated sexual experience?	(6.32)	(5.32)		(7.51)	(6.57)		(3.41)	(1.0)	
6. How many times have you had a sexual	1.24	1.18	0.10	1.27	1.67	–0.49	0.86	0.35	1.17
encounter you engaged in willingly but later regretted?	(2.94)	(5.31)		(2.77)	(6.56)		(1.88)	(0.81)	
7. How many partners have you had sex with?	5.51	3.28	1.78	6.08	3.33	1.53	4.39	3.50	0.46
	(11.53)	(6.38)		(13.21)	(7.25)		(7.91)	(4.77)	
8. How many people have you had sex	2.17	1.49	0.74	1.99	1.98	0.11	2.61	0.70	1.09
with that you know but are not involved in any sort of relationship with?	(6.32)	(5.40)		(5.66)	(6.65)		(7.81)	(1.13)	
9. How many times have you had sex with	2.07	1.54	0.66	1.89	1.86	0.03	5.18	2.92	0.93
someone you don't know well or just met?	(5.27)	(5.65)		(5.31)	(6.75)		(0.78)	(0.65)	
10. How many times have you or your partner	9.24	6.82	0.77	11.43	7.05	0.96	11.33	15.79	–0.78
used alcohol or drugs before or during sex?	(22.19)	(17.94)		(26.18)	(19.58)		(1.71)	(3.53)	
11. How many times have you had sex with	4.45	6.82	–0.80	4.16	4.14	0.01	9.03	22.49	–0.84
someone who has had many sexual partners?	(15.71)	(16.38)		(15.53)	(13.46)		(1.36)	(5.03)	
12. How many partners have you had sex with	1.33	1.18	0.21	1.24	1.64	–0.42	4.40	0.66	1.06
that you didn't trust?	(4.06)	(5.72)		(4.02)	(7.09)		(0.66)	(0.17)	

*HCs, healthy controls*.

* *p = 0.05. Bold values mean statistical significance after correcting for multiple testing*.

In order to determine whether or not ADHD symptomatology was associated with sexual risk-taking behavior, we calculated a sexual risk score by summing up the raw values of the variables shown in Table 5. This was possible because in any variable higher values corresponded to more risk-taking behavior. The associations between the total sexual risk score and ADHD symptomatology measured with the SR-WRAADDS are shown in Table 6.

**Table 6:** Association between ADHD symptomatology and overall risk score using the SRS.

**Sex Risk Survey (SRS)**	**Whole sample**	**Women**	**Men**
**sum score**	**(*n* = 139)**	**(*n* = 89)**	**(*n* = 44)**
SR-WRAADDS sum score	0.15	0.23^*^	0.08
Attention difficulties	−0.01	0.01	0.07
Hyperactivity	0.04	0.12	−0.13
Temper	0.21^*^	0.22^*^	0.34^*^
Affective lability	0.06	0.13	−0.14
Emotional over-reactivity	0.03	0.13	−0.08
Disorganization	0.06	0.11	−0.02
Impulsivity	0.24^**^	0.28^**^	0.21
Oppositional symptoms	0.23^**^	0.30^**^	0.15
Academic problems	0.02	0.06	−0.04
Social attitudes	0.08	0.12	0.06

* *p = 0.05*;

** *p < 0.01; Bold values mean statistical significance after correcting for multiple testing*.

In women, ADHD symptomatology in general was positively associated with sexual risk-taking behavior. Furthermore, a significant positive correlation was found between temper, impulsivity, and oppositional symptoms and sexual risk-taking. In men, only temper was significantly associated with sexual risk-taking, while all other scales did not show statistically significant associations.

### Sexual Dysfunctions

Men with ADHD did not report more or less frequently about erectile dysfunctions during masturbation or sexual intercourse, about problems with delayed or early ejaculation and were just as satisfied with their orgasm as men without ADHD (see Table 7). In women with or without ADHD, also no differences occurred in the frequency of low orgasm satisfaction, sexual pain or missing orgasm (see Table 8).

**Table 7:** Frequency of sexual dysfunctions in men with ADHD compared to men without ADHD using the SBQ-G.

	**ADHD**	**HCs**	**χ^2^**
	**(*n* = 42)**	**(*n* = 21)**	
Erectile dysfunction masturbation	1 (2.4%)	2 (9.5%)	1.58
Erectile dysfunction intercourse	8 (19.0%)	5 (23.8%)	0.19
Delayed ejaculation	4 (10.3%)	5 (23.8%)	1.97
Early ejaculation	12 (30.8%)	3 (15.0%)	1.73
Low orgasm satisfaction	13 (31.0%)	5 (23.8%)	0.35

*HCs, healthy controls*.

^*^p = 0.05;

^**^p < 0.01.

**Table 8:** Frequency of sexual dysfunctions in women with ADHD compared to women without ADHD using the SBQ-G.

	**ADHD**	**HCs**	**χ^2^**
	**(*n* = 84)**	**(*n* = 39)**	
Low orgasm satisfaction	19 (22.6%)	6 (15.4%)	0.86
Sexual pain	5 (6.8%)	3 (8.1%)	0.06
Failure to achieve orgasm	22 (25.3%)	13 (33.3%)	0.87

*HCs, healthy controls*.

^*^p = 0.05;

^**^p < 0.01.

Correlational analysis in ADHD participants showed only weak associations between sexual dysfunctions and ADHD symptoms. In women, only temper and affective lability showed a small but significant association with low orgasm satisfaction, while academic problems were positively related to the failure to reach an orgasm during masturbation or sexual intercourse and to sexual pain (see Table 9). In men, hyperactivity showed a medium correlation with erectile dysfunction during sexual intercourse, whereas affective lability was negatively associated with delayed ejaculation. There were no other significant correlations in men with ADHD (see Table 10).

**Table 9:** Association between ADHD symptoms and sexual dysfunctions in women with ADHD using the SR-WRAADDS and the SBQ-G.

	**Failure to achieve orgasm**	**Low orgasm satisfaction**	**Sexual pain**
SR-WRAADDS sum score	0.07	0.13	0.17
Attention difficulties	0.14	0.04	0.20
Hyperactivity	−0.05	−0.01	0.16
Temper	0.04	0.24^*^	0.01
Affective lability	0.05	0.22^*^	0.16
Emotional over-reactivity	0.07	0.13	0.11
Disorganization	0.06	0.01	0.05
Impulsivity	−0.13	0.01	−0.08
Oppositional symptoms	−0.08	0.08	0.03
Academic problems	0.22^*^	0.15	0.27^*^
Social attitudes	0.16	0.14	0.22

* *p = 0.05*;

^**^p < 0.01; Bold values mean statistical significance after correcting for multiple testing.

**Table 10:** Association between ADHD symptoms and sexual dysfunctions in men with ADHD using the SR-WRAADDS and the SBQ-G.

	**Erectile dysfunction masturbation**	**Erectile dysfunction intercourse**	**Delayed ejaculation**	**Early ejaculation**	**No orgasm satisfaction**
SR-WRAADDS sum score	−0.14	0.13	−0.07	0.09	0.03
Attention difficulties	−0.15	−0.04	0.08	−0.07	−0.16
Hyperactivity	0.05	0.40^**^	−0.12	0.18	0.04
Temper	−0.21	0.09	−0.01	0.04	0.09
Affective lability	0.03	−0.22	−0.33^*^	−0.13	−0.14
Emotional over-reactivity	−0.14	−0.01	−0.21	−0.09	0.01
Disorganization	−0.04	−0.11	−0.14	−0.01	−0.13
Impulsivity	0.14	0.10	0.10	0.21	0.08
Oppositional symptoms	−0.20	0.05	0.05	−0.01	0.07
Academic problems	−0.18	0.27	0.02	0.04	−0.07
Social attitudes	−0.08	0.18	−0.01	0.20	0.15

* *p = 0.05*;

** *p < 0.01; Bold values mean statistical significance after correcting for multiple testing*.

## Discussion

The present study is among the first assessing a wide variety of self-reported sexual behaviors in a sample of adults with ADHD in comparison to non-ADHD adults from the general population. Furthermore, we evaluated the association between different facets of ADHD symptomatology and (problematic) sexual behaviors and analyzed differences between men and women with ADHD.

In summary, we found no differences regarding risky sexual behaviors and sexual dysfunctions in the comparisons between the ADHD and the non-ADHD groups. Nevertheless, participants with ADHD reported significantly more hypersexual behaviors in a sub-clinical level, including higher HBI-19 total scores and higher scores on the HBI-19 subscales coping, consequences and control than participants without an ADHD. In the comparisons separated by gender, both women and men with ADHD showed significant higher HBI-19 sum scores and significant higher scores on its subscale control. However, we found no significant difference in the number of participants in the ADHD and non-ADHD groups, who were at or above the pre-defined HBI-19 cut-off value of 53 for the classification of an individual as hypersexual.

Usually going along with a higher number of sexual partners, there are other risky sexual behaviors in adults with ADHD. ADHD individuals usually show an earlier initiation of sexual activity and sexual intercourse, have more partner pregnancies during adolescence, use condoms during sexual intercourse less often, are more frequently diagnosed with a sexually transmitted infection, and use alcohol and drugs during sexual contacts more frequently compared to adults without ADHD (27, 32, 34). Strikingly, the results of the present study could not confirm this pattern. Although a wide range of risky sexual behaviors were assessed, we did not find any differences between individuals with and without ADHD except for men with ADHD being more likely to leave a social event with someone they have just met and women with ADHD being younger at their first sexual experience compared to their non-ADHD counterparts. However, previous research also suggested that risky sexual behaviors in individuals with ADHD occur especially during adolescence and thus it could be possible that adults with ADHD do not show more risky sexual behaviors anymore (37).

In samples from the general population but also in clinical samples, a relation between a higher impulsivity and more risky sexual behaviors was repeatedly found (61–63). In the present study, in women with ADHD the amount of risky sexual behaviors was indeed positively correlated with impulsivity, suggesting that impulsivity is a risk factor for risky sexual behaviors in adults with ADHD as well. Furthermore, it was proposed that conduct problems during childhood and adolescence might also be a relevant moderator of the relationship between ADHD and risky sexual behaviors (29, 64). We also found a significant correlation between risky sexual behaviors and oppositional symptoms at least in adult women with ADHD. Conduct problems are usually associated with more increased impulsive behaviors (65–67). Thus, it could be possible that women with ADHD showing risky sexual behaviors are especially prone to additionally show violations of rules and social norms in other areas. However, this suggestion is speculative and has to be treated cautiously.

In both men and women with ADHD, the temper subscale was also positively correlated with risky sexual behaviors. On the one side, frequent emotional outbursts also show a close relation to oppositional or conduct problems (68) and could thus be seen as further support for the above stated interaction between risky sexual behaviors and conduct problems. However, previous research has also found that risky sexual behaviors are used as a dysfunctional form of emotion regulation in clinical and non-clinical populations (69, 70) and our results suggest that this could account for ADHD individuals as well. Furthermore, previous research suggested that risky sexual behaviors as a behavioral expression of emotion regulation appears to be a relevant risk factor for sexual victimization (71, 72). In accordance to other previous studies (73–75), we found a high rate of almost 20% of previous sexually abusive experiences in our ADHD sample, and almost 25% in women with ADHD, showing that this might indeed be a relevant problem which should be addressed in clinical interventions aiming at improving emotion dysregulation.

While some studies have found a considerable prevalence of ADHD in samples of individuals reporting about hypersexual behaviors (39, 76–78), to our knowledge only one previous empirical study has looked into the prevalence of hypersexual behaviors in ADHD individuals so far (42). In that study between 5 and 12% of the ADHD men and 2% of the ADHD women fulfilled the predefined criteria of hypersexuality. However, it must be criticized that Bijlenga and colleagues (42) used a questionnaire to assess hypersexuality that has not been validated so far. In the present study, both men and women with ADHD had significantly higher total scores on the HBI-19, the worldwide probably most widely used questionnaire to assess hypersexual behaviors. These differences remained on a sub-clinical level though. When comparing the number of ADHD and non-ADHD individuals who were above the predefined cut-off for pathological hypersexuality, no group differences occurred. However, we found considerably higher numbers concerning pathological hypersexual behaviors in our ADHD participants compared to the study of Bjilenga and colleagues (42). Furthermore, an association between the intensity of ADHD symptoms and hypersexuality was previously found (40), which could be confirmed based on the present results. Whereas, previous studies suggested that ADHD patients meeting the criteria of the inattentive subtype were most likely to show hypersexual behaviors (76, 79), in the present study, hypersexual behaviors were closely associated with the SR-WRAADDS subscales impulsivity, temper, affective lability, emotional over-reactivity and oppositional symptoms, at least in women. Theoretical models and empirical studies have linked impulsivity as well as problems with emotion regulation to the emergence of hypersexual behaviors (80–83). Increased impulsivity might lead to an inability to resist sexual impulses regardless of whether they might be personally harmful and to act on a sexual urge with little forethought (84). This might also explain why both hypersexuality as well as sexual risk-taking were closely related to impulsivity in the present sample, at least in ADHD women. Concerning emotional dysregulation, previous studies have shown that hypersexual behaviors are used by many individuals as a dysfunctional behavior to relieve negative affect, to cope with affective symptoms, or to face adverse life events (85, 86). Therefore, it appears obvious that those ADHD patients showing more signs of emotional dysregulation also report about more hypersexual behaviors.

In both men and women with ADHD hypersexual behaviors were also related to the SR-WRAADDS subscale social attitudes. This subscale includes items like “difficult, strained relationship with life partner”, “problems making friends and maintaining friendships, “few social contacts.”, or “cool relationships with life partners”. It could be hypothesized that individuals scoring high on this subscale view sexuality in general and sexual relationships in specific rather unemotional and as a mere satisfaction of their own needs without caring much about the feelings and needs of the sexual partner. This would also explain why the HBI-19 subscale coping was the only one that was actually not related to the social attitudes subscale, underscoring that in these individuals' hypersexual behaviors cannot be seen as a means of dysfunctional emotion regulation. Moreover, it is known that fear of intimacy is increased in ADHD patients and correlates with the number of ADHD symptoms as well as with biographical experiences of devaluation and rejection and with risky sexual behavior, particularly among the inattentive subtype of ADHD, pointing that the inability for mutual dialogue, attachment and support could be linked to risky and hypersexual behaviors as well (87).

Although previous studies suggested that women as well as men with ADHD report more frequently about sexual dysfunctions compared to non-affected individuals from the general population (42, 44), no significant differences were found in the present study. In women with ADHD, low orgasm satisfaction was related to higher scores in temper and affective lability, again suggesting that especially problems with emotion regulation could lead to more sexual problems. However, only a small correlation was found, and these subscales did not correlate with any other assessed sexual dysfunction and thus these results should be interpreted cautiously. Nevertheless, it is conceivable that having problems with emotion regulation could lead to difficulties getting emotionally involved with another person, which is probably in many cases a prerequisite for a higher orgasm satisfaction. In men with ADHD, an at least medium sized correlation between hyperactivity and erectile dysfunction during sexual intercourse was found. No other subscales showed a positive association with any kind of sexual dysfunction in men with ADHD, suggesting that at least in our sample, ADHD symptoms were less relevant for the occurrence of sexual dysfunctions, although previous research has found a different pattern (45). In a previous study (45), after controlling for anxiety/depression, no significant differences between ADHD and the control group regarding sexual dysfunction could be found anymore, suggesting that comorbid symptoms might play a more relevant role on sexual dysfunctions than ADHD specific symptomology.

Moreover, we did not find any differences in self-reported sexual orientation between individuals with and without ADHD. Corresponding to large epidemiological studies, in both groups a heterosexual orientation was reported most frequently, followed by a bisexual orientation, and a homosexual orientation (88). However, individuals with ADHD were clearly more likely to identify themselves as being bisexual compared to what has been suggested by previous research with samples from the general population. However, corresponding to previous research, although individuals with ADHD reported being heterosexual almost as often as non-ADHD individuals in our study, significantly more ADHD patients reported about previous same-sex sexual experiences (88). In another study, among homo-/bisexual men in a male sample of young men presenting for mandatory military conscription, an elevated prevalence rate of 10.8% for ADHD in the past 12 months was found (89), possibly suggesting a correlation between ADHD and sexual orientation fluidity. Furthermore, previous research suggested that individuals with ADHD have more sexual partners than non-ADHD individuals (12, 30, 32), which might result in a tendency to be more open to different kinds of sexual experiences. However, speaking against this conclusion, individuals with and without ADHD did not significantly differ in the present study concerning the number of previous sexual partners, although this comparison showed a trend in approaching the pre-defined level of significance (*p* = 0.08). Based on previous and our findings, it could also be possible that due to a lower overall satisfaction within sexual and romantic relationships (90, 91), ADHD patients tend to try out not only more, but also different kinds of sexual partners until they find a partner they really get along with. It is also conceivable, that this pattern in individuals with ADHD emerges out of boredom and a stronger need for self-stimulation, curiosity in general, impulsivity, an increased sex drive, or due to fewer constraints by social norms.

Because information gathered for the present study were based on self-report only and the chosen approach was rather exploratory, the results are limited and should be interpreted cautiously. Further, other limitations should be addressed: We did not calculate the sample size a priori and our final sample was quite small, thus generalizability of the results is limited. Especially the sample of non-ADHD participants was quite small. We could only include 21 men without ADHD, which could have hampered the detection of gender specific group differences Further, although all ADHD participants have specified that they were diagnosed by a psychologist or psychiatrist, one cannot be sure that this was actually the case. However, the ADHD individuals had significantly higher SR-WRAADDS total scores and scored significantly higher on every subscale, underscoring that they showed a more pronounced ADHD symptomatology than the non-ADHD individuals. Another important limitation of the present study is related again to possible sampling biases: given we recruited participants from the general population using ADHD specific Internet fora and other online channels, namely different social media platforms and mailing lists, it might have contributed to a selection of highly motivated individuals in the clinical and control group. Although we excluded cases that did not complete the whole questionnaire, the intensity of ADHD symptomatology did not differ between those ADHD participants who completed the whole questionnaire and those who did not. Furthermore, our results could also be limited by the fact that especially participants with a higher interest in sexuality-related issues or participants with per se more sexual problems were more likely to participate in the present study, because our study call included the information, that the online survey would assess sexual behaviors and sexual problems. This could have led to an overestimation of hypersexual behaviors and sexual dysfunctions. Such sampling biases can affect the internal validity of an analysis by leading to inaccurate estimations of relationships between variables and the external validity of an analysis, thus the results from a biased sample may not generalize to the population. Furthermore, although we assessed how many individuals were currently treated with medication, we did not assess the kind of medication our ADHD individuals were treated with. Since previous research has shown that especially stimulants could have an influence on sexual functioning, the specific medication should be considered in future studies assessing sexuality related issues in patients with ADHD (45, 92, 93). In addition, we did not consider psychiatric co-morbidity in our study, which is a common phenomenon in adults with ADHD (e.g. anxiety, depression, substance use disorders) and might also have an impact on sexual functions.

Future studies should assess the perception concerning sexuality-related issues of the intimate partners of patients with ADHD. To fulfill this purpose, future studies could for example use an interview design assessing sexual experiences of both the patients and their partners. Due to a higher likelihood of risky sexual behaviors in patients with ADHD, they might also be at an increased risk of sexual victimization, a highly relevant issue that has so far been mainly neglected by previous research. Finally, as symptoms of emotion regulation seem to be especially relevant concerning the occurrence of sexual problems in patients with ADHD, future studies should focus more strongly on this relationship.

## Conclusions

A healthy sexuality is of utmost importance for overall well-being (94). However, sexuality-related issues are rarely addressed during medical consultations (95, 96). Based on the results of the present survey it could be shown that patients with ADHD show certain peculiarities in their sexual functioning, underscoring that in these patients there is a particular need that sexuality-related issues should be routinely addressed during clinical visits. This accounts even more since patient surveys have shown that the majority of patients hopes that their health care provider asks them about sexuality-related issues (97). By considering these recommendations, one could contribute to a more holistic treatment of individuals with ADHD, thereby increasing their overall quality of life.

## Data Availability Statement

The raw data supporting the conclusions of this article will be made available by the authors, without undue reservation.

## Ethics Statement

The studies involving human participants were reviewed and approved by the Ethics Committee of the Psychological Chamber of the University Medical Center Hamburg-Eppendorf, Germany. The patients/participants provided their written informed consent to participate in this study.

## Author Contributions

PGH, DT, DS, SB, and WR: conceptualization and methodology. PGH, DT, and WR: formal analysis. PGH, DT, and LB: investigation. WR: resources. PGH, DT, and LB: data curation. PGH, DT, and WR: writing—original draft preparation. PGH, WR, DT, SB, and PR-J: writing—review and editing. PGH, DT, DS, LB, PR-J, and WR: visualization. WR: administration. All authors have read and agreed to the published version of the manuscript.

## Conflict of Interest

The authors declare that the research was conducted in the absence of any commercial or financial relationships that could be construed as a potential conflict of interest.

## Publisher's Note

All claims expressed in this article are solely those of the authors and do not necessarily represent those of their affiliated organizations, or those of the publisher, the editors and the reviewers. Any product that may be evaluated in this article, or claim that may be made by its manufacturer, is not guaranteed or endorsed by the publisher.
